# Diet can alter the cost of resistance to a natural parasite in *Caenorhabditis elegans*

**DOI:** 10.1002/ece3.9793

**Published:** 2023-02-09

**Authors:** Juliana Jiranek, Amanda Gibson

**Affiliations:** ^1^ Department of Biology University of Virginia Charlottesville Virginia USA

**Keywords:** *Caenorhabditis*, context dependence, costs of resistance, defense, host–parasite interactions, *Nematocida*, QTLs, trade‐off

## Abstract

Resistance to parasites confers a fitness advantage, yet hosts show substantial variation in resistance in natural populations. Evolutionary theory indicates that resistant and susceptible genotypes can coexist if resistance is costly, but there is mixed evidence that resistant individuals have lower fitness in the absence of parasites. One explanation for this discrepancy is that the cost of resistance varies with environmental context. We tested this hypothesis using *Caenorhabditis elegans* and its natural microsporidian parasite, *Nematocida ironsii*. We used multiple metrics to compare the fitness of two near‐isogenic host genotypes differing at regions associated with resistance to *N. ironsii*. To quantify the effect of the environment on the cost associated with these known resistance regions, we measured fitness on three microbial diets. We found that the cost of resistance varied with both diet and the measure of fitness. We detected no cost to resistance, irrespective of diet, when fitness was measured as fecundity. However, we detected a cost when fitness was measured in terms of population growth, and the magnitude of this cost varied with diet. These results provide a proof of concept that, by mediating the cost of resistance, environmental context may govern the rate and nature of resistance evolution in heterogeneous environments.

## Graphical abstract

Evolutionary theory suggests that the cost of parasite resistance promotes the coexistence of resistant and susceptible individuals. However, empirical tests do not consistently find a cost of resistance. Using a nematode–microsporidian sytem, we found support for the hypothesis that costs of resistance vary with the environmental context in which they are measured.

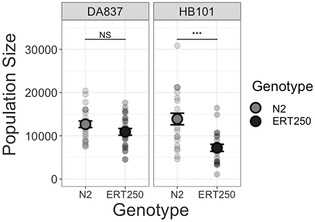

## 1 INTRODUCTION

In natural populations, individuals show tremendous variation in their ability to resist parasites (Little, 2002). This variation appears counterintuitive: if resistance to disease increases an individual's fitness, beneficial resistance alleles should sweep through a population (Smith & Haigh, 1974; Stephan, 2019). A common explanation for this variation in resistance is that resistant hosts pay a cost, such that resistance covaries negatively with other components of fitness. This negative covariance could be functional (i.e., pleiotropy) or arise from the physical linkage of resistance genes with other genes impacting fitness (Brady et al., 2019; Lazzaro & Little, 2009). When resistance is costly, resistant individuals have relatively low fitness in the absence of parasite exposure (Duffy et al., 2012; Little, 2002; Schwenke et al., 2016). A large body of theory has shown that if resistance is costly, resistant, and susceptible genotypes can stably coexist in a population (Antonovics & Thrall, 1994; Boots & Haraguchi, 1999; Gillespie, 1975). In support of this hypothesis, many empirical studies have shown that resistant hosts have reduced fitness relative to susceptible hosts in the absence of a parasite (Biere & Antonovics, 1996; Kraaijeveld et al., 2002; Tian et al., 2003).

Despite the compelling theoretical and empirical support for costs of resistance, many experiments have not found evidence of costs, suggesting that they are not universal (Gupta et al., 2016; Parker, 1990; Penley et al., 2018). A potential reason that costs are not always observed is that they may only manifest in certain environments. Several studies have found that resistance is only costly when individuals experience limited resources (Boots, 2011; Hernandez & Koskella, 2019; Kraaijeveld & Godfray, 1997). Building on this work, we sought to test whether environmental context can create variation in the cost of genomic regions that confer parasite resistance.

**FIGURE 1:**
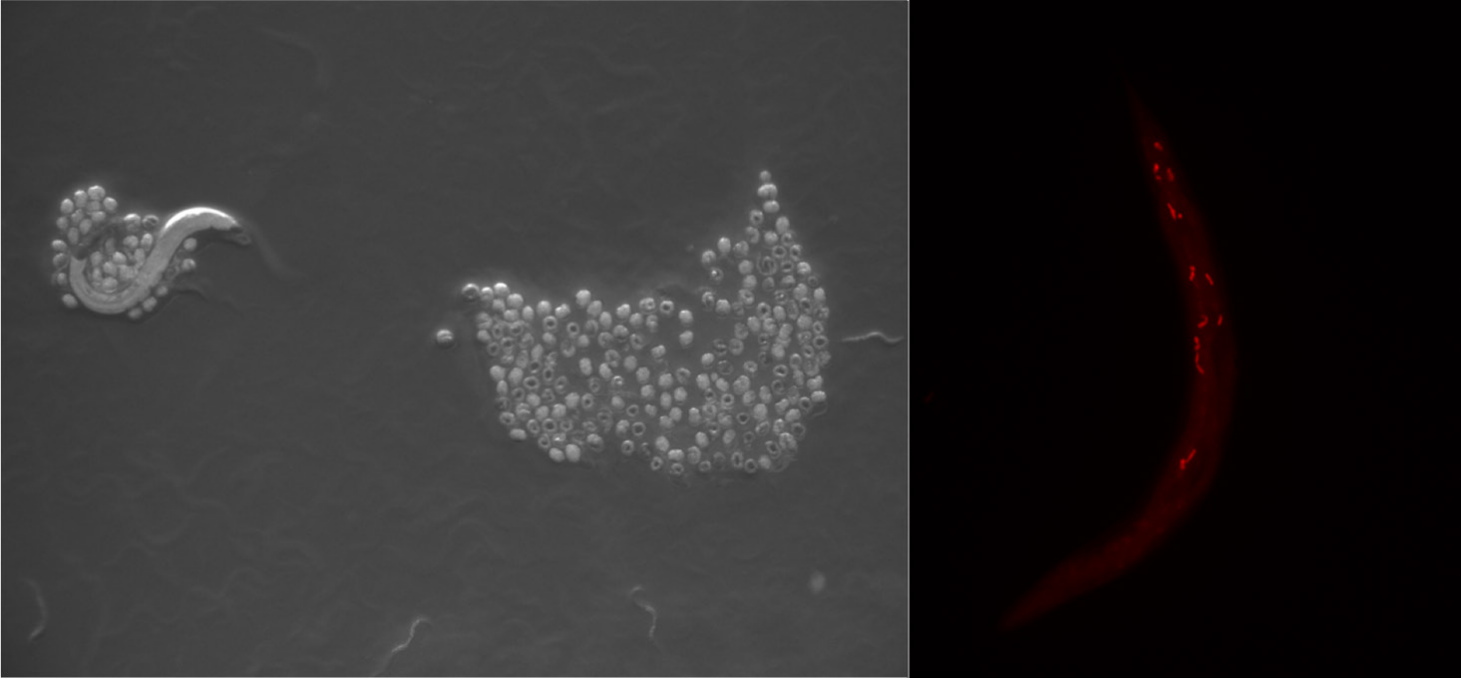
An adult *C. elegans* nematode surrounded by eggs and larvae (left) and a nematode infected with the intestinal parasite *N. ironsii*, which is labeled with red fluorescence (right). Photos by Anne Janisch.

To assess variation in the fitness costs of parasite resistance across environments, we used the nematode *Caenorhabditis elegans*, which shows variation in resistance to its natural, environmentally‐transmitted microsporidian parasite, *Nematocida ironsii* (Figure 1). Two quantitative trait loci (QTL) located on chromosomes II and V are associated with resistance in wild lineages of *C. elegans* (Balla et al., 2015; Mok et al., 2022). To test for a cost associated with these resistance QTL, we used near‐isogenic lines (NILs) of *C. elegans* carrying either the resistant or susceptible variants on a shared genetic background (Balla et al., 2015). We compared the fitness of the resistant and susceptible NILs in the absence of *N. ironsii* through assays of individual fecundity and population expansion. Because bacterial diet is a key component of *C. elegans* natural environment and is strongly linked to population growth (Samuel et al., 2016), we compared fitness across three strains of *Escherichia coli* that represent qualitatively distinct diets for *C. elegans*. We found that the cost of resistance varied with both diet and the fitness measure used.

## 2 METHODS

### 2.1 Study system

Forward genetic screens with *C. elegans* have demonstrated that mutations that increase *Nematocida* resistance can simultaneously decrease other fitness components (Reddy et al., 2017, 2019). We build on this prior work by evaluating the costs of genomic regions associated with *Nematocida* resistance in natural lineages of *C. elegans* that vary in their resistance capabilities. The Hawaiian lineage CB4856 can clear *N. ironsii* infection from the intestinal epithelial cells during the first larval (L1) stage of development (Balla et al., 2015), while the lab‐adapted lineage N2 cannot. Two QTL located on chromosomes II and V explain a large fraction of this variation in resistance (Balla et al., 2015). The resistant NIL, ERT 250, carries these resistance QTL on an N2 genetic background and was generated by the Troemel lab at UCSD (Balla et al., 2015). We acquired the susceptible host genotype, N2, from the *Caenorhabditis* Genetics Center (CGC).

The size and location of the resistance QTL are detailed in Balla et al. (2015). Briefly, the region on chromosome II spans the peak of the *rami‐1* QTL and is less than 1 Mb in size. *rami‐1* explains 15% of the variation in the *N. ironsii* clearance phenotype between N2 and CB4856. The 1.2 Mb region on chromosome V spans the peak of the *rami‐4* QTL, which explains 12% of the variation in clearance (Balla et al., 2015). The specific genes that confer resistance remain unknown. Each region includes many genes that would likely be inherited together under natural conditions because outcrossing only occurs in an estimated 1% of *C. elegans* matings (Andersen & Rockman, 2022; Barrière & Félix, 2005). Neither QTL encompasses the *npr‐1* locus, which has previously been reported to drive large fecundity differences between N2 and CB4856 (Andersen et al., 2014).

To simulate the variable microbial environments that *C. elegans* experiences in the wild, we varied diet qualitatively. We selected three different strains of the standard bacterial food *Escherichia coli*: OP50, DA837, and HB101. These strains differ with regard to palatability to hosts, effects on host behavior, and effects on host growth rates. The strain DA837 is considered a low‐quality food for *C. elegans* because its cells clump together, making it more difficult for hosts to ingest (Brooks et al., 2009). When raised on this food, hosts roam, indicating a lack of satiety (You et al., 2008). The strain HB101 is considered a high‐quality food because individuals grow faster and attain larger body sizes compared with OP50 (So et al., 2011). The strain OP50 is the standard lab food for *C. elegans* and is considered to be of intermediate quality relative to DA837 and HB101. Consistent with these differences, in preference tests, hosts choose HB101 over OP50, and OP50 over DA837 (Shtonda & Avery, 2006). There are, however, no substantial differences in lifespan or fecundity between individuals raised on HB101 and OP50 (Brooks et al., 2009).

Bacterial cultures were grown in LB inoculated with a single colony. NGM plates were seeded with bacteria at a concentration of 5 × 10^−7^ CFU/ml and incubated overnight at 28°C.

### 2.2 Fecundity assay

We conducted two assays to assess different components of fitness across diet conditions. First, to determine whether the resistant genotype has lower fecundity than the susceptible genotype, we counted the number of offspring produced by ERT250 and N2 hosts raised on the three bacterial diets. For each genotype, we isolated eggs using a standard bleach wash (Porta‐de‐la‐Riva et al., 2012), then added approximately 100 eggs per plate to DA837‐, OP50‐, or HB101‐seeded 100 mm plates. We allowed hosts to reach the fourth larval (L4) stage at 20°C then moved hosts individually to 35 mm plates seeded with the same bacteria as their original plate (*n* = 60 hosts, with one host per 35 mm plate; 10–14 hosts per genotype*food combination). We moved each host to a new plate every day for six days, at which point reproduction had finished. Plates with eggs were incubated at 20°C for 24 h to hatch, then we counted viable offspring. We censored data from hosts that were lost or suffered damage unrelated to the test conditions (*n* = 4). Data from an additional three hosts were excluded from the analysis because of reporting errors.

### 2.3 Population expansion assay

To test whether resistant populations grow at a slower rate than susceptible populations, we measured the size of resistant and susceptible populations after a standardized period of expansion. To ensure sufficient replication for each treatment, we limited this assay to DA837 and HB101. For both N2 and ERT250, we picked L4 hosts individually onto 100 mm plates seeded with either DA837 or HB101 (*n* = 100 hosts, with one host per 100 mm plate; 25 hosts per genotype*food combination). Plates were monitored daily. The populations expanded for six days (~2–3 generations), at which point we collected entire populations in M9 buffer, diluted the populations to 14.5 ml, and counted six 20 μl aliquots for each population. The total population size could be estimated by multiplying these aliquot counts by 725. Replicate populations were removed from the analysis if the original host died before producing offspring (*n* = 2) or if there was an error during the washing and resuspension steps (*n* = 4).

### 2.4 Statistical analysis

We performed all analyses in R Version 1.4.1106 (R Core Team, 2021). To test for variation in fitness, we used the lme4 package (Bates et al., 2015) to fit generalized linear models or mixed models (GLMM). For the fecundity data, we analyzed both variations in lifetime fecundity (the total number of offspring summed over all days) and variations in the number of offspring per day, which can reveal differences in reproductive timing. For the fecundity models, we found evidence of overdispersion under the Poisson distribution, so we assumed a negative binomial distribution. We compared multiple candidate models including host genotype, food type, and their interaction as predictors. For the daily number of offspring, we also included the day of observation and its interaction with each of the aforementioned predictors, as well as a random effect to account for repeated measures of the same host. For the population expansion data, we fit Poisson GLMMs, including host genotype, food type, and their interaction as predictors of hosts per aliquot, plus a random effect for the replicate population to account for repeated measures. For all analyses, we used AIC scores to compare candidate models.

## 3 RESULTS

**FIGURE 2:**
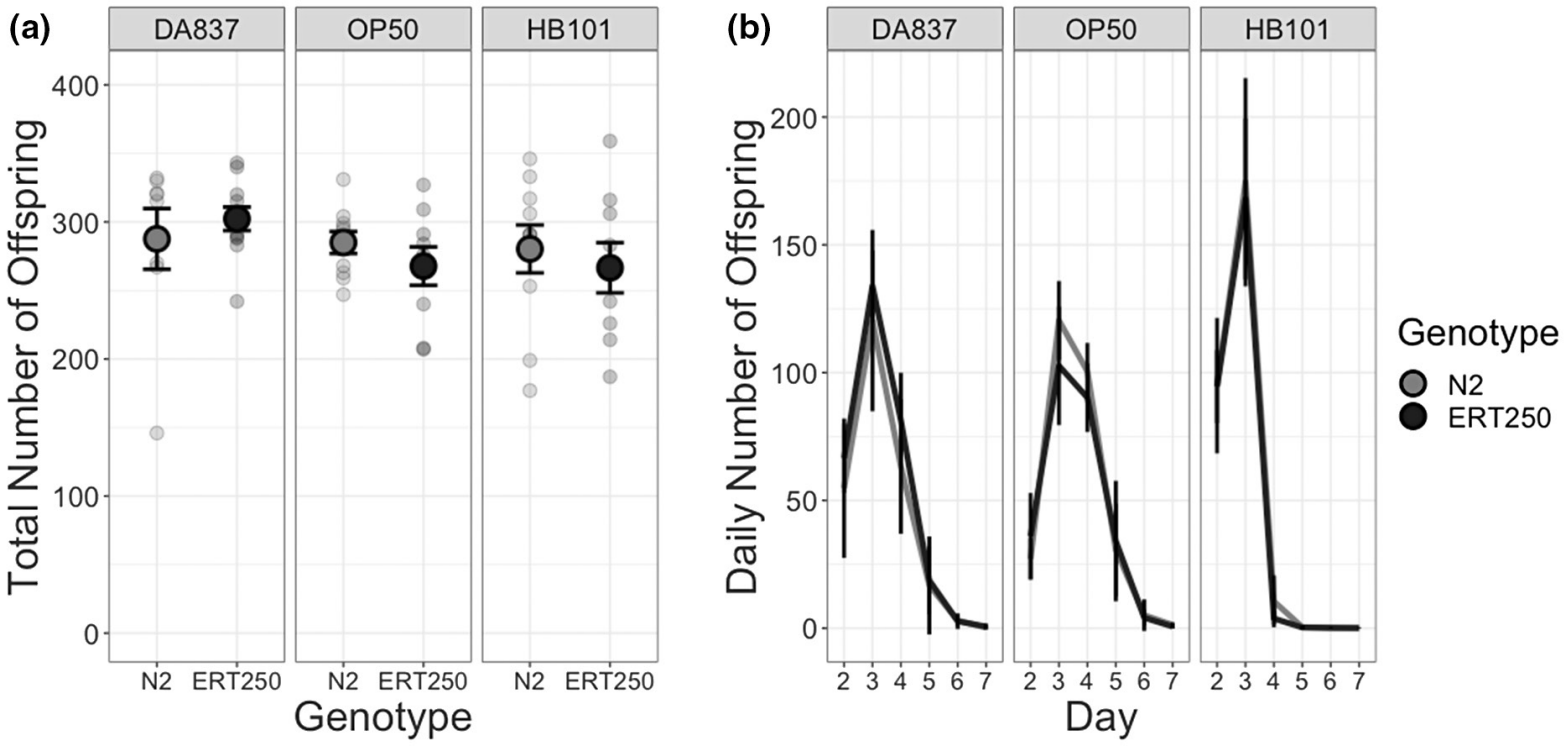
Diet affects reproductive timing but not the cost of resistance as measured by individual fecundity. (a) Lifetime and (b) daily fecundity of susceptible (N2: light gray) and resistant hosts (ERT250: dark gray) across three bacterial diets. Lifetime fecundity did not vary with genotype or diet. The timing of reproduction did vary with diet, with hosts producing earlier on HB101. Error bars show the standard error of the mean.

To test for environmentally‐driven variation in costs of two QTL that confer parasite resistance, we compared the fitness of susceptible and resistant NILs through assays of fecundity and population expansion on three microbial diets. In the fecundity assay, we found that susceptible and resistant hosts did not differ significantly in lifetime fecundity, regardless of diet (Figure 2a, Table 1, *p* > .05 for all comparisons). On DA837, resistant individuals produced slightly more offspring (302 ± 9, standard error of the mean) than susceptible individuals (288 ± 22). On the other two diets, resistant individuals produced about five percent fewer offspring than susceptible individuals (OP50: 268 ± 14 vs. 285 ± 8; HB101 267 ± 18 vs. 280 ± 18), but these differences were not statistically significant. Individuals produced slightly more offspring on DA837 (296 ± 10) than on the other food types (OP50: 277 ± 8; HB101: 274 ± 12), but these differences also were not significant (Table 1b).

**TABLE 1:** Summary of analyses for lifetime fecundity^a^

(*a*) *Model comparison*				
Model		AIC	ΔAIC	Weight
**Null**		606.36	0.00	0.59
Food		607.91	1.55	0.27
Genotype + Food		609.68	3.32	0.11
Genotype*Food		612.38	6.02	0.03
(*b*) *Summary of 2nd best model*				
Model: Total Offspring ~ Food				
Predictor	Level	Coefficient ± SE	z score	*p* value
(Intercept)		5.69 ± 0.04	149.88	<.001***
Food	OP50	−0.07 ± 0.05	−1.25	.211
	HB101	−0.08 ± 0.05	−1.46	.145

^a^ Each row corresponds to a different statistical model, and the columns show the terms, the AIC score, ΔAIC, and Akaike weight for each model. The best model (lowest AIC and ΔAIC, highest weight) is indicated in bold.

***p < .001.

Similarly, susceptible and resistant hosts did not differ in the timing of reproduction, as determined by daily counts of viable offspring (Figure 2b, Table 2). However, the timing of reproduction did vary with food type (best model: Daily offspring count ~ Day*Food type). Hosts raised on HB101 produced over 99% of their offspring by day 3, while hosts on OP50 and DA837 had produced only 52% and 71% of their offspring, respectively, by day 3.

**TABLE 2:** Summary of analyses for daily offspring production

(*a*) *Model comparison*				
Model		AIC	ΔAIC	Weight
Day*Food		2574.53	0.00	0.64
Day*Food + Genotype		2576.50	1.97	0.24
Day*Food + Genotype*Food		2577.73	3.20	0.13
Additional candidate models had no support				
(*b*) *Best model summary*				
Model: Offspring ~ Day*Food + (1|host individual)				
Predictor	Level	Coefficient ± SE	z score	*p* value
(Intercept)		4.13 ± 0.16	25.82	<.001***
Day	3	0.71 ± 0.20	3.51	<.001***
	4	0.09 ± 0.21	0.41	.685
	5	−1.44 ± 0.23	−6.29	<.001***
	6	−3.21 ± 0.25	−12.79	<.001***
	7	−4.97 ± 0.38	−13.07	<.001***
Food	OP50	−0.68 ± 0.23	−2.89	.004**
	HB101	0.43 ± 0.23	1.85	.065
Day:Food	3:OP50	0.56 ± 0.30	1.87	.061
	4:OP50	1.03 ± 0.31	3.37	<.001***
	5:OP50	1.40 ± 0.32	4.37	<.001***
	6:OP50	1.18 ± 0.35	3.35	<.001***
	7:OP50	1.47 ± 0.50	2.96	.003**
	3:HB101	−0.14 ± 0.30	−0.48	.633
	4:HB101	−2.80 ± 0.32	−8.84	<.001***
	5:HB101	−4.21 ± 0.49	−8.60	<.001***
	6:HB101	−3.69 ± 0.78	−4.73	<.001***
	7:HB101	−2.61 ± 1.09	−2.40	.017*

*p < .05; **p < .01; ***p < .001.

**FIGURE 3:**
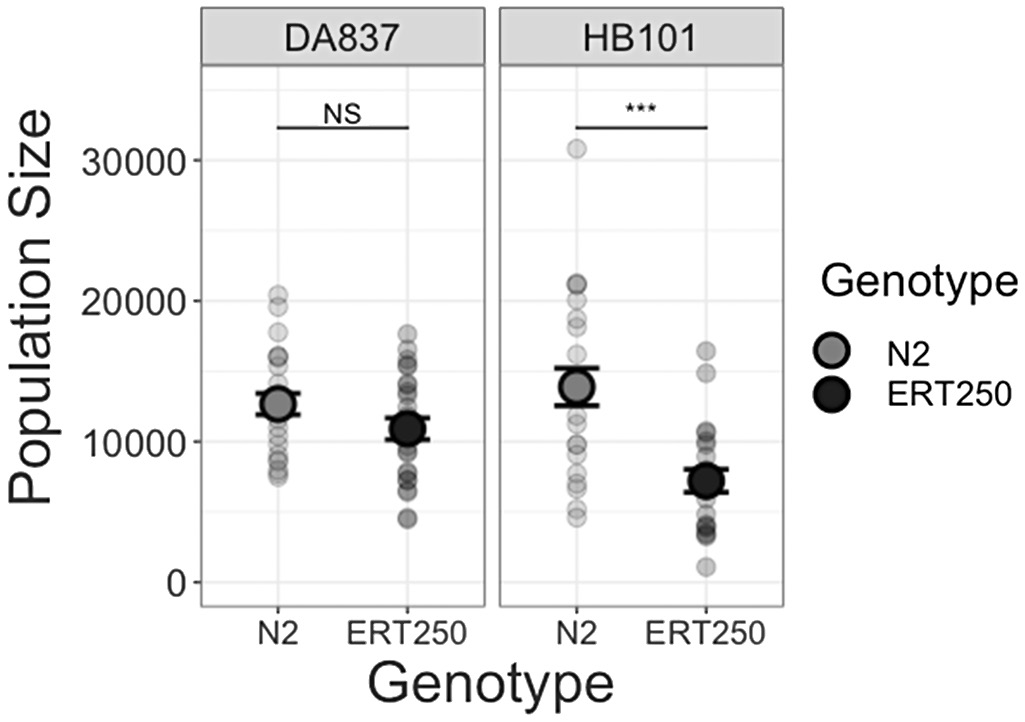
The resistant genotype has reduced population size on HB101 after six days of expansion. Population size was calculated as 725 times the average number of hosts across six 20 μl aliquots per replicate population. Susceptible N2 is in light gray and resistant ERT250 is in dark gray. Error bars show the standard error of the mean.

We then compared the size of populations of the resistant and susceptible genotypes after six days of proliferation. Population size varied with the interaction of food type and genotype (Figure 3, Table 3). On HB101, the susceptible genotype achieved a mean population size of 13,885 ± 1333, nearly twice that of the resistant genotype (7212 ± 815) (Tukey test, *Z* = 5.30, *p* < .0001, Figure 3). This result remained when the outlier in the HB101*N2 group was excluded from the analysis (Table 3c). On DA837, mean population size of the susceptible genotype was 16% larger than that of the resistant genotype, but this difference was not statistically significant (N2: 12,667 ± 756, vs. ERT250: 10,909 ± 766) ($\chi^{2}$, *Z* = −1.41, *p* = .159).

**TABLE 3:** Summary of analyses for population expansion.

(*a*) *Model comparison*				
Model		AIC	ΔAIC	Weight
Genotype*Food		3381.32	0.00	**0.94** ^a^
Genotype + Food		3387.27	5.94	0.05
Genotype		3390.66	9.34	0.01
Food		3403.55	22.23	0.00
(*b*) *Summary of Best Model*				
Model: Count ~ Genotype * Food + (1| Replicate Population)				
Predictor	Level	Coefficient ± SE	z score	*p* value
(Intercept)		2.82 ± 0.09	32.63	<.001***
Genotype	Resistant	−0.17 ± 0.12	−1.41	.159
Food	HB101	0.03 ± 0.12	0.28	.782
Genotype:Food	Resistant: HB101	−0.51 ± 0.18	−2.88	.004**

^a^ Each row corresponds to a different statistical model, and the columns show the terms, the AIC score, ΔAIC, and Akaike weight for each model. The best model (lowest AIC and ΔAIC, highest weight) is indicated in bold.

*p < .05; **p < .01; ***p < .001.

## 4 DISCUSSION

We compared near‐isogenic *C. elegans* lines to test whether the cost of resistance to the natural parasite *Nematocida ironsii* varies with environmental context. We found evidence that the cost of resistance varies with both fitness metric and host diet. We detected no cost of resistance when measuring fitness as individual fecundity (Figure 2). However, when we allowed populations to proliferate for multiple generations, the resistant genotype reached smaller population sizes than the susceptible genotype, indicating a reduced population growth rate (Figure 3).

We only detected this cost of resistance on the diet HB101, which past literature had indicated was the highest quality of our three diets. This result contrasts with prior studies that found a cost of resistance only in food‐limited contexts (Boots, 2011; Kraaijeveld & Godfray, 1997). Because we only detected this cost over multiple generations of population growth, we hypothesize that the resistant genotype has an increased sensitivity to density, manifesting as a reduced birth rate, increased development time, and/or increased death rate as density increases (Andersen et al., 2014; Seidel & Kimble, 2011; Wong et al., 2020). We found that individuals raised on HB101 reproduced earlier than their counterparts on other diets (Figure 2b), consistent with faster development times on HB101 reported in the literature (So et al., 2011). Thus, though total offspring numbers and population sizes on HB101 were ultimately not greater than on the other diets, the first wave of reproduction on HB101 would have rapidly increased density early in the population expansion assay. Moreover, by day six of population growth, populations on HB101 were expected to have older, larger individuals than populations on DA837. The body volume of adult *C. elegans* is more than four times that of the first larval stage (Uppaluri & Brangwynne, 2015), so differences in age structure can create major differences in density when measured by body mass rather than numbers of individuals. Thus, if the resistant genotype has increased density sensitivity, we expect this cost to be more apparent on the HB101 diet than on DA837.

We chose to measure fitness through population expansion because of the ecology of *C. elegans*. In the wild, one to a few nematodes colonize a transient microbial resource, reproduce rapidly, predominantly via self‐fertilization, then disperse to a new patch (Félix & Braendle, 2010; Richaud et al., 2018; Sloat et al., 2022). These microbial resources are highly ephemeral, so demes that reach large population sizes quickly are expected to produce more migrants to colonize new patches. Within a patch, a *C. elegans* lineage reaches large population sizes quickly by having relatively high individual fecundity, early reproduction, and reduced sensitivity to density (Hodgkin & Barnes, 1991). Together, these components of population growth determine a genotype's contribution to the dispersal pool and its long‐term persistence in the metapopulation. Therefore, density sensitivity may represent a fitness cost of resistance that has real implications for resistance evolution in wild populations of *C. elegans*.

Many tests of costs of resistance in animal‐disease systems have used lineages generated under artificial selection for resistance (Faria et al., 2015; Gupta et al., 2016; Penley et al., 2018) or sampled from natural populations (Auld et al., 2013; Graham et al., 2010). The use of NILs is much rarer, although studies in plant systems have demonstrated the power of this approach (Tian et al., 2003; reviewed in Bergelson & Purrington, 1996). These studies find that the costs of resistance may be more apparent when there is greater control over the genetic background, thereby reducing the masking effect of compensatory mutations (Bergelson & Purrington, 1996). Our study used NILs that differ at two genomic regions associated with a parasite clearance phenotype in the wild *C. elegans* genotype CB4856. These genomic regions were also independently identified in association with *N. ironsii* resistance in the Spanish *C. elegans* lineage JU1440 (Mok et al., 2022). Thus, in evaluating the fitness costs of these resistance regions, our work may provide general insights into the evolution of microsporidia resistance in *C. elegans*. These two genomic regions contain many genes, so we are unable to distinguish between pleiotropy and linkage as drivers of the observed population growth rate difference between susceptible and resistant genotypes (Fernandes et al., 2021; Gianola et al., 2015). However, outcrossing is very rare in *C. elegans* (Barrière & Félix, 2005), so pleiotropy and linkage may have similar consequences for the evolution of resistance (Andersen & Rockman, 2022).

In the wild, the substrates on which *C. elegans* live harbor diverse microbial communities (Samuel et al., 2016; Schulenburg & Félix, 2017). Our results demonstrate that the cost of resistance varies with microbial diet, suggesting that the heterogeneous environments that *C. elegans* experiences in the wild may constrain resistance evolution. The benefit of resistance may also vary with microbial diet: nematodes acquire *Nematocida* parasites while foraging, so exposure varies with foraging rate, which in turn varies with diet (Shtonda & Avery, 2006). Thus, our study provides initial support for the idea that variation in resistance to *Nematocida* in natural *C. elegans* populations may be maintained by environmental heterogeneity that modulates the costs, and potentially the benefits, of resistance.

## AUTHOR CONTRIBUTIONS

**Juliana Jiranek:** Conceptualization (supporting); data curation (equal); formal analysis (lead); investigation (lead); project administration (supporting); visualization (lead); writing – original draft (lead); writing – review and editing (equal). **Amanda Gibson:** Conceptualization (lead); formal analysis (supporting); funding acquisition (lead); investigation (supporting); methodology (lead); project administration (lead); resources (lead); supervision (lead); validation (supporting); visualization (supporting); writing – original draft (supporting); writing – review and editing (equal).

## CONFLICT OF INTEREST

The authors declare no conflicts of interest.

## FUNDING INFORMATION

This work was supported by funding from the National Institute of General Medical Sciences (R35 GM137975‐01) and the Jeffress Trust Awards Program in Interdisciplinary Research.

### OPEN RESEARCH BADGES

This article has earned Open Data, Open Materials and Preregistered Research Design badges. Data, materials and the preregistered design and analysis plan are available at [[insert provided URL(s) on the Open Research Disclosure Form]].

## Data Availability

The study's raw data and analysis scripts are archived on Dryad (doi:10.5061/dryad.2v6wwpzsv) and on the corresponding author's GitHub repository (https://github.com/jjiranek/cost).

## References

[ece39793-bib-0001] Andersen, E. C. , Bloom, J. S. , Gerke, J. P. , & Kruglyak, L. (2014). A variant in the neuropeptide receptor *npr*‐1 is a major determinant of *Caenorhabditis elegans* growth and physiology. PLoS Genetics, 10, e1004156. 10.1371/journal.pgen.1004156 24586193 PMC3937155

[ece39793-bib-0002] Andersen, E. C. , & Rockman, M. V. (2022). Natural genetic variation as a tool for discovery in *Caenorhabditis* nematodes. Genetics, 220, iyab156. 10.1093/genetics/iyab156 35134197 PMC8733454

[ece39793-bib-0003] Antonovics, J. , & Thrall, P. H. (1994). The cost of resistance and the maintenance of genetic polymorphism in host‐pathogen systems. Proceedings of the Royal Society of London. Series B: Biological Sciences, 257, 105–110.

[ece39793-bib-0004] Auld, S. K. J. R. , Penczykowski, R. M. , Housley Ochs, J. , Grippi, D. C. , Hall, S. R. , & Duffy, M. A. (2013). Variation in costs of parasite resistance among natural host populations. Journal of Evolutionary Biology, 26, 2479–2486. 10.1111/jeb.12243 24118613

[ece39793-bib-0005] Balla, K. M. , Andersen, E. C. , Kruglyak, L. , & Troemel, E. R. (2015). A wild *C. elegans* strain has enhanced epithelial immunity to a natural microsporidian parasite. PLoS Pathogens, 11, e1004583. 10.1371/journal.ppat.1004583 25680197 PMC4334554

[ece39793-bib-0006] Barrière, A. , & Félix, M. A. (2005). High local genetic diversity and low outcrossing rate in *Caenorhabditis elegans* natural populations. Current Biology, 15, 1176–1184. 10.1016/j.cub.2005.06.022 16005289

[ece39793-bib-0007] Bates, D. , Maechler, M. , Bolker, B. , & Walker, S. (2015). Fitting linear mixed‐effects models using lme4. Journal of Statistical Software, 64, 1–48. 10.18637/jss.v067.i01

[ece39793-bib-0008] Bergelson, J. , & Purrington, C. B. (1996). Surveying patterns in the cost of resistance in plants. The American Naturalist, 148, 536–558.

[ece39793-bib-0009] Biere, A. , & Antonovics, J. (1996). Sex‐specific costs of resistance to the fungal pathogen *Ustilago violacea* (*Microbotryum violaceum*) in *Silene alba* . Evolution, 50, 1098–1110. 10.1111/j.1558-5646.1996.tb02350.x 28565272

[ece39793-bib-0010] Boots, M. (2011). The evolution of resistance to a parasite is determined by resources. The American Naturalist, 178, 214–220. 10.1086/660833 21750385

[ece39793-bib-0011] Boots, M. , & Haraguchi, Y. (1999). The evolution of costly resistance in host‐parasite systems. The American Naturalist, 153, 359–370. 10.1086/303181 29586625

[ece39793-bib-0012] Brady, S. P. , Bolnick, D. I. , Angert, A. L. , Gonzalez, A. , Barrett, R. D. H. , Crispo, E. , Derry, A. M. , Eckert, C. G. , Fraser, D. J. , Fussmann, G. F. , Guichard, F. , Lamy, T. , McAdam, A. G. , Newman, A. E. M. , Paccard, A. , Rolshausen, G. , Simons, A. M. , & Hendry, A. P. (2019). Causes of maladaptation. Evolutionary Applications, 12, 1229–1242. 10.1111/eva.12844 31417611 PMC6691215

[ece39793-bib-0013] Brooks, K. K. , Liang, B. , & Watts, J. L. (2009). The influence of bacterial diet on fat storage in *C. elegans* . PLoS One, 4, e7545. 10.1371/journal.pone.0007545 19844570 PMC2760100

[ece39793-bib-0014] Duffy, M. A. , Ochs, J. H. , Penczykowski, R. M. , Civitello, D. J. , Klausmeier, C. A. , & Hall, S. R. (2012). Ecological context influences epidemic size and parasite‐driven evolution. Science, 335, 1636–1638. 10.1126/science.1215429 22461614

[ece39793-bib-0015] Faria, V. G. , Martins, N. E. , Paulo, T. , Teixeira, L. , Sucena, É. , & Magalhães, S. (2015). Evolution of *drosophila* resistance against different pathogens and infection routes entails no detectable maintenance costs. Evolution, 69, 2799–2809. 10.1111/evo.12782 26496003

[ece39793-bib-0016] Félix, M. A. , & Braendle, C. (2010). The natural history of *Caenorhabditis elegans* . Current Biology, 20, R965–R969. 10.1016/j.cub.2010.09.050 21093785

[ece39793-bib-0017] Fernandes, S. B. , Zhang, K. S. , Jamann, T. M. , & Lipka, A. E. (2021). How well can multivariate and univariate GWAS distinguish between true and spurious pleiotropy? Frontiers in Genetics, 11, 1747. 10.3389/fgene.2020.602526 PMC7873880 33584799

[ece39793-bib-0018] Gianola, D. , de los Campos, G. , Toro, M. A. , Naya, H. , Schön, C. C. , & Sorensen, D. (2015). Do molecular markers inform about pleiotropy? Genetics, 201, 23–29. 10.1534/genetics.115.179978 26205989 PMC4566266

[ece39793-bib-0019] Gillespie, J. H. (1975). Natural selection for resistance to epidemics. Ecology, 56, 493–495. 10.2307/1934983

[ece39793-bib-0020] Graham, A. L. , Hayward, A. D. , Watt, K. A. , Pilkington, J. G. , Pemberton, J. M. , & Nussey, D. H. (2010). Fitness correlates of heritable variation in antibody responsiveness in a wild mammal. Science, 330, 662–665. 10.1126/science.1194878 21030656

[ece39793-bib-0021] Gupta, V. , Venkatesan, S. , Chatterjee, M. , Syed, Z. A. , Nivsarkar, V. , & Prasad, N. G. (2016). No apparent cost of evolved immune response in *drosophila melanogaster* . Evolution, 70, 934–943. 10.1111/evo.12896 26932243

[ece39793-bib-0022] Hernandez, C. A. , & Koskella, B. (2019). Phage resistance evolution in vitro is not reflective of in vivo outcome in a plant‐bacteria‐phage system. Evolution, 73, 2461–2475. 10.1111/evo.13833 31433508

[ece39793-bib-0023] Hodgkin, J. , & Barnes, T. M. (1991). More is not better: Brood size and population growth in a self‐fertilizing nematode. Proceedings of the Royal Society of London. Series B: Biological Sciences, 246, 19–24. 10.1098/rspb.1991.0119 1684664

[ece39793-bib-0024] Kraaijeveld, A. R. , Ferrari, J. , & Godfray, H. C. J. (2002). Costs of resistance in insect‐parasite and insect‐parasitoid interactions. Parasitology, 125, S71–S82. 10.1017/S0031182002001750 12622330

[ece39793-bib-0025] Kraaijeveld, A. R. , & Godfray, H. C. J. (1997). Trade‐off between parasitoid resistance and larval competitive ability in *Drosophila melanogaster* . Nature, 389, 278–280. 10.1038/38483 9305840

[ece39793-bib-0026] Lazzaro, B. P. , & Little, T. J. (2009). Immunity in a variable world. Philosophical Transactions of the Royal Society, B: Biological Sciences, 364, 15–26. 10.1098/rstb.2008.0141 PMC2666692 18926975

[ece39793-bib-0027] Little, T. J. (2002). The evolutionary significance of parasitism: Do parasite‐driven genetic dynamics occur ex silico? Journal of Evolutionary Biology, 15, 1–9. 10.1046/j.1420-9101.2002.00366.x

[ece39793-bib-0028] Mok, C. , Xiao, M. A. , Wan, Y. C. , Zhao, W. , Ahmed, S. M. , Luallen, R. , & Reinke, A. W. (2022). High‐throughput phenotyping of *C. elegans* wild isolates reveals specific resistance and susceptibility traits to infection by distinct microsporidia species. 10.1101/2022.06.20.496912 PMC10030041 36893187

[ece39793-bib-0029] Parker, M. A. (1990). The pleiotropy theory for polymorphism of disease resistance genes in plants. Evolution, 44, 1872–1875. 10.2307/2409515 28567805

[ece39793-bib-0030] Penley, M. J. , Greenberg, A. B. , Khalid, A. , Namburar, S. R. , & Morran, L. T. (2018). No measurable fitness cost to experimentally evolved host defence in the *Caenorhabditis elegans–Serratia marcescens* host–parasite system. Journal of Evolutionary Biology, 31, 1976–1981. 10.1111/jeb.13372 30187979

[ece39793-bib-0031] Porta‐de‐la‐Riva, M. , Fontrodona, L. , Villanueva, A. , & Cerón, J. (2012). Basic *Caenorhabditis elegans* methods: Synchronization and observation. JoVE (Journal of Visualized Experiments), 64, e4019. 10.3791/4019 PMC3607348 22710399

[ece39793-bib-0032] R Core Team . (2021). R: A language and environment for statistical computing. R Core Team.

[ece39793-bib-0033] Reddy, K. C. , Dror, T. , Sowa, J. N. , Panek, J. , Chen, K. , Lim, E. S. , Wang, D. , & Troemel, E. R. (2017). An intracellular pathogen response pathway promotes proteostasis in *C. elegans* . Current Biology, 27, 3544–3553. 10.1016/j.cub.2017.10.009 29103937 PMC5698132

[ece39793-bib-0034] Reddy, K. C. , Dror, T. , Underwood, R. S. , Osman, G. A. , Elder, C. R. , Desjardins, C. A. , Cuomo, C. A. , Barkoulas, M. , & Troemel, E. R. (2019). Antagonistic paralogs control a switch between growth and pathogen resistance in *C. elegans* . PLoS Pathogens, 15, e1007528. 10.1371/journal.ppat.1007528 30640956 PMC6347328

[ece39793-bib-0035] Richaud, A. , Zhang, G. , Lee, D. , Lee, J. , & Félix, M. A. (2018). The local coexistence pattern of selfing genotypes in *Caenorhabditis elegans* natural metapopulations. Genetics, 208, 807–821. 10.1534/genetics.117.300564 29242287 PMC5788539

[ece39793-bib-0036] Samuel, B. S. , Rowedder, H. , Braendle, C. , Félix, M. A. , & Ruvkun, G. (2016). *Caenorhabditis elegans* responses to bacteria from its natural habitats. Proceedings of the National Academy of Sciences of the United States of America, 113, E3941–E3949. 10.1073/pnas.1607183113 27317746 PMC4941482

[ece39793-bib-0037] Schulenburg, H. , & Félix, M. A. (2017). The natural biotic environment of *Caenorhabditis elegans* . Genetics, 206, 55–86. 10.1534/genetics.116.195511 28476862 PMC5419493

[ece39793-bib-0038] Schwenke, R. A. , Lazzaro, B. P. , & Wolfner, M. F. (2016). Reproduction–immunity trade‐offs in insects. Annual Review of Entomology, 61, 239–256. 10.1146/annurev-ento-010715-023924 PMC5231921 26667271

[ece39793-bib-0039] Seidel, H. S. , & Kimble, J. (2011). The oogenic germline starvation response in *C. elegans* . PLoS One, 6, e28074. 10.1371/journal.pone.0028074 22164230 PMC3229504

[ece39793-bib-0040] Shtonda, B. B. , & Avery, L. (2006). Dietary choice behavior in *Caenorhabditis elegans* . Journal of Experimental Biology, 209, 89–102. 10.1242/jeb.01955 16354781 PMC1352325

[ece39793-bib-0041] Sloat, S. A. , Noble, L. M. , Paaby, A. B. , Bernstein, M. , Chang, A. , Kaur, T. , Yuen, J. , Tintori, S. C. , Jackson, J. L. , Martel, A. , Salome Correa, J. A. , Stevens, L. , Kiontke, K. , Blaxter, M. , & Rockman, M. V. (2022). *Caenorhabditis* nematodes colonize ephemeral resource patches in neotropical forests. Ecology and Evolution, 12, e9124. 10.1002/ece3.9124 35898425 PMC9309040

[ece39793-bib-0042] Smith, J. M. , & Haigh, J. (1974). The hitch‐hiking effect of a favourable gene. Genetics Research, 23, 23–35. 10.1017/S0016672300014634 4407212

[ece39793-bib-0043] So, S. , Miyahara, K. , & Ohshima, Y. (2011). Control of body size in *C. elegans* dependent on food and insulin/IGF‐1 signal. Genes to Cells, 16, 639–651. 10.1111/j.1365-2443.2011.01514.x 21501345

[ece39793-bib-0044] Stephan, W. (2019). Selective sweeps. Genetics, 211, 5–13. 10.1534/genetics.118.301319 30626638 PMC6325696

[ece39793-bib-0045] Tian, D. , Traw, M. B. , Chen, J. Q. , Kreitman, M. , & Bergelson, J. (2003). Fitness costs of R‐gene‐mediated resistance in Arabidopsis thaliana. Nature, 423, 74–77. 10.1038/nature01588 12721627

[ece39793-bib-0046] Uppaluri, S. , & Brangwynne, C. P. (2015). A size threshold governs *Caenorhabditis elegans* developmental progression. Proceedings of the Royal Society B: Biological Sciences, 282, 20151283. 10.1098/rspb.2015.1283 PMC4632629 26290076

[ece39793-bib-0047] Wong, S. S. , Yu, J. , Schroeder, F. C. , & Kim, D. H. (2020). Population density modulates the duration of reproduction of *C. elegans* . Current Biology, 30, 2602–2607. 10.1016/j.cub.2020.04.056 32442457 PMC7343598

[ece39793-bib-0048] You, Y. , Kim, J. , Raizen, D. M. , & Avery, L. (2008). Insulin, cGMP, and TGF‐β signals regulate food intake and quiescence in *C. elegans*: A model for satiety. Cell Metabolism, 7, 249–257. 10.1016/j.cmet.2008.01.005 18316030 PMC3786678

